# Peer support for discharge from inpatient mental health care versus care as usual in England (ENRICH): a parallel, two-group, individually randomised controlled trial

**DOI:** 10.1016/S2215-0366(21)00398-9

**Published:** 2022-02

**Authors:** Steve Gillard, Stephen Bremner, Akshaykumar Patel, Lucy Goldsmith, Jacqueline Marks, Rhiannon Foster, Rosaleen Morshead, Sarah White, Sarah L Gibson, Andrew Healey, Mike Lucock, Shalini Patel, Julie Repper, Miles Rinaldi, Alan Simpson, Michael Ussher, Jessica Worner, Stefan Priebe, ENRICH trial study group

**Affiliations:** a School of Health Sciences, City, University of London, London, UK; b Department of Primary Care and Public Health, Brighton and Sussex Medical School, Brighton, UK; c Pragmatic Clinical Trials Unit, Queen Mary, University of London, London, UK; d Unit for Social and Community Psychiatry, Queen Mary, University of London, London, UK; e Population Health Research Institute, St George's, University of London, London, UK; f King's Health Economics, King's College London, London, UK; g Institute of Psychiatry, Psychology and Neuroscience, King's College London, London, UK; h Centre for Applied Research in Health, University of Huddersfield, Huddersfield, UK; i Adult Community Mental Health Team, South West London and St George's Mental Health NHS Trust, London, UK; j Strategy and Transformation, South West London and St George's Mental Health NHS Trust, London, UK; k Implementing Recovery through Organisational Change, Nottingham, UK; l Centre for Work and Mental Health, Nordland Hospital Trust, Bodø, Norway; m Institute for Social Marketing and Health, University of Stirling, Stirling, UK; n Together for Mental Wellbeing, London, UK

## Abstract

**Background:**

High numbers of patients discharged from psychiatric hospital care are readmitted within a year. Peer support for discharge has been suggested as an approach to reducing readmission post-discharge. Implementation has been called for in policy, however, evidence of effectiveness from large rigorous trials is missing. We aimed to establish whether peer support for discharge reduces readmissions in the year post-discharge.

**Methods:**

We report a parallel, two-group, individually randomised, controlled superiority trial, with trial personnel masked to allocation. Patients were adult psychiatric inpatients (age ≥18 years) with at least one previous admission in the preceding 2 years, excluding those who had a diagnosis of any organic mental disorder, or a primary diagnosis of learning disability, an eating disorder, or drug or alcohol dependency, recruited from seven state-funded mental health services in England. Patients were randomly assigned (1:1) to the intervention (peer support plus care as usual) or control (care as usual) groups by an in-house, online randomisation service, stratified by site and diagnostic group (psychotic disorders, personality disorders, and other eligible non-psychotic disorders) with randomly permuted blocks of randomly varying length to conceal the allocation sequence and achieve the allocation ratio. The peer support group received manual-based, one-to-one peer support, focused on building individual strengths and engaging with activities in the community, beginning during the index admission and continuing for 4 months after discharge, plus care as usual. Care as usual consisted of follow-up by community mental health services within 7 days of discharge. The primary outcome was psychiatric readmission 12 months after discharge (number of patients readmitted at least once), analysed on an intention-to-treat basis. All patients were included in a safety analysis, excluding those who withdrew consent for use of their data. The trial is registered with the ISRCTN registry, ISRCTN10043328. The trial was complete at the time of reporting.

**Findings:**

Between Dec 1, 2016, and Feb 8, 2019, 590 patients were recruited and randomly assigned, with 294 allocated to peer support (287 included in the analysis after withdrawals and loss to follow-up), and 296 to care as usual (291 in the analysis). Mean age was 39·7 years (SD 13·7; range 18–75). 306 patients were women, 267 were men, three were transgender, and two preferred not to say. 353 patients were White, 94 were Black, African, Caribbean, or Black British, 68 were Asian or Asian British, 48 were of mixed or multiple ethnic groups, and 13 were of other ethnic groups. In the peer support group, 136 (47%) of 287 patients were readmitted at least once within 12 months of discharge. 146 (50%) of 291 were readmitted in the care as usual group. The adjusted risk ratio of readmission was 0·97 (95% CI 0·82–1·14; p=0·68), and the adjusted odds ratio for readmission was 0·93 (95% CI 0·66–1·30; p=0·68). The unadjusted risk difference was 0·03 (95% CI –0·11 to 0·05; p=0·51) in favour of the peer support group. Serious adverse events were infrequent (67 events) and similar between groups (34 in the peer support group, 33 in the care as usual group). Threat to life (self-harm) was the most common serious adverse event (35 [52%] of 67 serious adverse events). 391 other adverse events were reported, with self-harm (not life threatening) the most common (189 [48%] of 391).

**Interpretation:**

One-to-one peer support for discharge from inpatient psychiatric care, plus care as usual, was not superior to care as usual alone in the 12 months after discharge. This definitive, high-quality trial addresses uncertainty in the evidence base and suggests that peer support should not be implemented to reduce readmission post-discharge for patients at risk of readmission. Further research needs to be done to improve engagement with peer support in high-need groups, and to explore differential effects of peer support for people from different ethnic communities.

**Funding:**

UK National Institute for Health Research.

**Research in context**

**Evidence before this study**

We conducted a systematic review and meta-analysis of randomised controlled trials of one-to-one peer support for adults using mental health services. We searched the Cochrane Central Register of Controlled Trials, CINAHL Plus, Embase, Medline, and PsycINFO from their inception until June 13, 2019. Intervention search terms were “peer”, “consumer”, “survivor”, or “prosumer” adjacent to “support”, “supporter”, “provider”, “worker”, “specialist”, “consultant”, “tutor”, “educator”, “mentor”, “intervention”, “listener”, “mediator”, “counsellor”, “befriender”, or “therapist”. We excluded studies in which mental health was not the primary focus of the intervention, or peer support was not intentionally provided, one-to-one, or the primary means of delivering the intervention. The review was restricted to studies published in English. We retrieved 23 papers meeting criteria for the review, reporting 19 randomised controlled trials. 16 papers reporting 14 trials were included in the meta-analysis, considering a range of clinical, service use, and psychosocial outcomes. With relevance to this study, pooled results showed that there was a reduction in the relative risk of readmission of 14%, from 49% in the control group to 43% in the peer support group over a period ranging from 9 to 24 months post-randomisation; however, the result was not statistically significant and data for this outcome were retrieved from just five trials and 497 participants. Furthermore, trial quality was mixed with, of the 19 trials included in the review, seven at low-to-moderate risk of bias overall and eight at high risk of bias in one or more areas. The search was updated on Dec 17, 2020, identifying four additional trials, none of which measured psychiatric hospitalisation.

**Added value of this study**

This trial provides clear evidence that peer support for discharge from psychiatric inpatient care is not effective in reducing readmission in the year after discharge for patients with a high risk of readmission, compared with care as usual at discharge. This trial provides definitive, high-quality data that add clarity to an equivocal evidence base. The trial was indicative of a small-to-medium reduction in readmission for Black patients, and a small reduction for patients who were in receipt of peer support at least a minimum amount, with both findings of statistical significance. We found no evidence for an effect on psychosocial outcomes considered in our study, but we did not measure self-reported recovery or empowerment, for which a positive effect has been identified in systematic reviews.

**Implications of all the available evidence**

Evidence from studies of one-to-one peer support in a range of mental health service settings suggests peer support at discharge does not have a significant effect on subsequent hospitalisation, days in hospital, or severity of symptoms in the following year. Although no good evidence of cost-effectiveness is available, the implication for policy is that one-to-one peer support should not be commissioned in mental health services for patients at high risk of readmission with an expectation of a significant effect on clinical and service use outcomes. Further research is needed to clarify potential psychosocial benefits. More research also needs to be done to improve engagement with peer support in high-need groups, and to explore differential effects of peer support for people from different ethnic communities.

## Introduction

Discharge from psychiatric hospital treatment and the transition to outpatient care is, for many patients, a challenging period. Suicide rates in the first 3 months after discharge are approximately 100 times higher than in the general population,^1^ and readmission rates are high internationally, ranging from 33% at 3 months post-discharge^2^ to 41% at 1 year.^3^ The strongest predictor of early readmission is previous psychiatric hospitalisation,^4^ suggesting that patients with a history of admissions are in particular need of support at discharge. Some evidence suggests that follow-up by mental health services after discharge reduces readmissions, although what constitutes follow-up varies widely, and quality of studies is largely poor.^5^ A systematic review suggested that interventions specifically designed to support the transition from inpatient to community mental health care are feasible and likely to be cost-effective.^6^ Two of the studies included in the review incorporated peer support.^7^, ^8^

Peer support is rapidly being introduced into mental health care internationally, as advocated in national workforce plans^9^ and mental health policy,^10^ or within accredited health insurance schemes.^11^ Peer workers are people with experiences of living with mental health problems and using mental health services who are trained to provide various forms of support alongside usual mental health care in a range of clinical settings.^12^ Peer support specifically targeted at discharge and the transition to outpatient mental health care might mitigate potential harm resulting from disruption to clinical and social support and, thus, prevent readmissions.^13^ An observational study of peer support at discharge suggested a potential to reduce readmission rates,^14^ and a pilot randomised controlled trial indicated that conducting a large-scale trial of a transitional peer support intervention at discharge was feasible,^15^ but no definitive trials of peer support for discharge have been reported.

A systematic review and meta-analysis of trials evaluating one-to-one peer support in a range of mental health services showed a reduction in psychiatric hospital admissions of 14% for participants receiving peer support compared with those receiving care as usual.^16^ However, the data available were from only 497 participants, pooled from five studies, and the effect was not statistically significant. Earlier reviews also raised questions about the quality of the evidence base, including inadequate sequence generation and randomisation processes,^17^ no masking of assessors, and selective or incomplete reporting of outcomes.^18^ A further shortcoming of existing research is heterogeneity in the design of peer worker interventions, with many studies having no or poor description of the crucial ingredients of peer support,^18^ of the function of the peer worker, and of the training and organisational support for peer workers.^17^

Although peer support for discharge from psychiatric inpatient treatment is attracting considerable interest, high quality trials are needed to evaluate its effectiveness. Trials should specify the details of the peer worker interventions, including the organisational support provided to deliver peer support. The present study aimed to establish the effectiveness of a peer worker intervention to reduce psychiatric readmission following discharge. We hypothesised that patients receiving peer support for discharge, in addition to care as usual, would be significantly less likely to be readmitted in the year following discharge than patients receiving care as usual only.

## Methods

### Study design and participants

The study was a parallel, two-group, individually randomised, controlled superiority trial, with trial personnel (outcome assessors, data analysts) masked to the allocation of patients. The study took place in the inpatient and community mental health services of seven state mental health service providers in England (appendix p 13). At five sites, patients were recruited from two inpatient facilities, and at one site, recruitment was from three facilities; all of these sites were treated as single sites for stratification. A detailed trial protocol has been published.^19^ In an internal pilot of trial procedures, recruitment began at two sites, with stop-go criteria prespecified in the protocol, before commencing at the remaining sites. A process evaluation and fidelity study will be reported elsewhere. The study was approved by the UK National Research Ethics Service, Research Ethics Committee London—London Bridge (London, UK) on May 10, 2016, reference number 16/LO/0470.

All new admissions to participating adult acute inpatient wards were screened for eligibility. Inpatients were eligible if they had at least one previous psychiatric admission in the preceding 2 years, were aged 18 years or older, and were assessed by the ward clinical team as likely to be discharged within the next month and as having capacity to give written informed consent to participate in the research. Patients were excluded if they had a diagnosis of any organic mental disorder or had a primary diagnosis of eating disorders, learning disability, or drug or alcohol dependency as recorded in clinical notes (to avoid requiring an excessive range of specialist knowledge and skills from peer workers), or were assessed by the clinical team on the ward as presenting a current, substantial risk to a peer worker. Patients initially assessed as unlikely to be discharged within a month were rescreened weekly until they were assessed as likely to be discharged within a month, at which point full eligibility was determined.

Eligible patients were approached by a clinician on the ward and, if interested in participating in the study, given written information about the study. They were then approached by a member of the study team (research assistant), invited to give written informed consent to participate, and enrolled in the study. Peer workers were eligible to participate in the study if they had been trained and employed to provide the manual-based peer support for discharge delivered in the trial. All eligible peer workers at each study site were invited to give written informed consent to participate in the study.

### Randomisation and masking

After completion of baseline assessments, consenting patients were randomly assigned to the intervention (peer support plus care as usual) or control treatment (care as usual) groups in a 1:1 ratio. The randomisation sequence was generated by an in-house, online randomisation service, stratified by site and diagnostic group with randomly permuted blocks of randomly varying length to conceal the allocation sequence and achieve the allocation ratio. Site group had seven strata (the seven study sites). Diagnostic group had three strata based on the primary diagnosis of the patient for the index admission: psychotic disorders (ICD-10 diagnoses F20–29); personality disorders (ICD-10 diagnosis F60); and all other psychiatric diagnoses (excluding ICD-10 diagnoses F00–09, F10–19, F50, and F70–79). Allocation was generated and communicated to patients by a member of the study team (JM) not involved in assessments or data analysis. Measures to ensure protection and evaluation of masking of assessors were briefing patients before follow-up interview, self-report of the first two outcomes measures in a closed envelope, and completion of a masking form by the study team member collecting follow-up data, and are detailed in the study protocol.^19^ Study team members analysing data were masked to allocation.

#### Procedures

Patients in the intervention group received the peer support intervention, delivered one-to-one by a designated peer worker, a discharge information pack, and care as usual at discharge. Patients were assigned a peer worker after treatment allocation and before discharge. Peer workers were assigned by a peer worker coordinator employed at each site who also provided support and supervision to the peer worker team and had experience of working as a peer supporter. Peer workers were matched to the patient by gender when specifically requested by the patient or felt to be appropriate by the clinical team.

The peer support intervention, including peer worker training programme (provided by the peer worker coordinator supported by clinical staff and others with relevant expertise at each site), is specified in a handbook and described in detail in the trial protocol.^19^ Patients in the intervention group were offered one or more face-to-face contacts with their peer worker in hospital before discharge and, once discharged, one meeting per week with the peer worker for 10 weeks, followed by three meetings over 6 weeks (one meeting per fortnight). The entire intervention lasted 4 months. Meetings were flexible in length, typically ranging from 60–90 mins, supplemented by phone calls and text messages. Initial meetings focused on building a relationship, with subsequent meetings making flexible use of the skills and tools covered in the peer worker training. The emphasis of the peer support was enabling the patient to access available social support, rather than the peer worker directly providing support. Peer workers could attend discharge and care planning meetings and appointments with care professionals at the patient's request.

The peer worker intervention is underpinned by a peer support principles framework^20^ and an empirically grounded change model.^21^ The handbook provides peer worker and peer worker coordinator role descriptions, specifies the support and supervision peer workers receive, and details preparation sessions for clinical teams providing care to patients in the intervention group in the trial. The training programme lasted about 8 weeks in total and comprised eight, 6-h training sessions, plus employment support, hospital visits, and structured feedback. Training covers guidance and practice for peer workers in using their own experience-based knowledge and use of a range of structured tools and exercises focused on building individual strengths and engaging with activities in the community (eg, personal asset mapping, goal setting, and discharge, recovery, and crisis planning).

The discharge information pack provides information about potentially useful statutory services and community services (not-for-profit). Patients were given a copy of the pack at treatment allocation and peer workers were able to make use of the pack. Care as usual after discharge from inpatient psychiatric care is mandated nationally in England as follow-up by community mental health services within 7 days of discharge.^22^

Patients in the control group received care as usual and a copy of the discharge information pack to control for any effect of access to information alone on outcomes.

Data were collected at baseline, and at 4 months (within 120–180 days) and 12 months (per the electronic patient record) after discharge from the index admission. After written consent was provided and before allocation to groups, a researcher collected baseline data from all patients using a structured face-to-face interview, which was repeated at 4 months. All information collected during the interviews are detailed in the protocol.^19^ Mental health service use data were extracted from the electronic patient record at each site at baseline and 12 months by site information management staff masked to patient allocation. The staff used a pro forma designed by trial personnel to be compatible with the management information system at each study site, indicating patients using their EPR identifier, and securely transferred data to the study team via secure servers.

#### Outcomes

The primary outcome for the trial was psychiatric inpatient readmission (number of patients readmitted at least once) in the 12 months after discharge from the index admission, including both involuntary and voluntary admissions. Secondary outcomes assessed at baseline and 4 months via the structured interviews were: subjective quality of life self-rated with the Manchester Short Assessment of Quality of Life (MANSA);^23^ social inclusion self-reported on the Objective Social Outcomes Index (SIX);^24^ hope for the future, self-rated on the Herth Hope Index (HHI);^25^ and psychiatric symptom levels, observer-rated on the Brief Psychiatric Rating Scale (BPRS).^26^ Additionally, strength of the patient's social network, assessed at 4 months as a secondary outcome, was self-reported using the Social Contacts Assessment^27^ with use of data on both the frequency and quality of social contacts in the preceding week. Other prespecified secondary outcomes, all collected at baseline for the 12 months before index admission and at 12 months post-discharge, were total number of psychiatric inpatient admissions (any type, voluntary, involuntary); days in hospital (across all psychiatric admissions); use of accident and emergency services for a psychiatric emergency, measured as number of episodes of liaison psychiatry contact; and number of contacts with crisis resolution and home treatment teams. Time to first readmission, measured in days post-discharge from the index admission, was also assessed as a secondary outcome.

Adherence to the intervention was assessed with a structured online survey completed by peer workers following each contact. Number, type (face-to-face or telephone), and duration of contacts with the peer worker, pre-discharge and post-discharge, were collected for each patient in the peer support group. All adverse events including serious adverse events, as defined in the trial protocol,^19^ were recorded and followed up until the end of the 12-month follow-up by site principle investigators (who also decided whether serious adverse events were related to trial treatments and whether events were unexpected).

### Choice of primary measure

We selected our primary outcome because high rates of readmission, including repeat readmissions, in the year after discharge are persistent in mental health care, and are in part indicative of inadequate support in the transition back to community.^2^, ^3^, ^5^, ^6^

### Statistical analysis

We required a sample size of 530 patients, allocated on a 1:1 ratio, to detect a reduction of 12% in readmission (from 34% to 22%) in the intervention group compared with the care as usual group, with 80% power at the 5% significance level. 34% was the mean 1-year readmission rate according to clinical activity data from the first 6 months of 2012 at three participating sites. This calculation allowed for clustering by peer worker in the intervention group only^28^ and, in the absence of any published estimate, assumed an intracluster correlation coefficient of 0·05 with an average cluster size of ten patients. We inflated the sample size by 10% to allow for missing primary outcome data at follow-up,^29^ resulting in a final sample size of 590.

Baseline characteristics are summarised for each treatment group by the mean and SD or median and IQR for continuous variables as appropriate, and the number and percentage for categorical variables.

All analyses were conducted according to the intention-to-treat (ITT) principle, meaning that all randomly assigned patients with a recorded outcome for primary or secondary outcomes were included in the analysis, and analysed according to the group to which they were allocated. We also estimated the complier average causal effect (CACE) for the intervention on the primary outcome^30^ (defining compliers as patients who had at least two peer worker meetings, at least one of which was in the community following discharge). Patients who withdrew consent for their data to be included in the analysis were excluded from all analyses, including safety analyses. All included patients were analysed for safety.

For analysis of the primary outcome and each secondary outcome we present: number of patients in each analysis, by treatment group; a summary statistic of the outcome (eg, number [%]), by treatment group; estimated treatment effect and corresponding 95% CI; and two-sided p value. For all analyses, a significance level of 5% was used. Analysis was conducted with Stata (version 16).

We planned to analyse the primary outcome using a mixed-effects logistic regression model with patients clustered by peer worker in the intervention group and patients being their own cluster in the control group.^28^ However, owing to a near-zero intracluster correlation, the model did not converge, so instead we fitted a single-level model ignoring clustering. The model was adjusted for the stratification variables (site and diagnostic group) and for prespecified baseline covariates that, based on existing research, were likely to be predictive of the primary outcome (ie, number of psychiatric admissions in the 12 months before the index admission and ethnicity),^2^, ^3^, ^4^ entered into the model as fixed effects. The analysis of the primary outcome was conducted using a logistic regression model which gives an estimated adjusted odds ratio (OR) and 95% CI. The adjusted risk ratio (RR) and its 95% CI were derived from this model by running the margins command in Stata. It is not possible to obtain an adjusted risk difference from such a model. Subgroup analyses for the primary outcome were prespecified and used the same model as the primary analysis, adjusting for the covariates used in the primary analysis and an interaction term between randomisation and the subgroup variable of interest: ethnicity (any Black ethnicity, all other ethnicities); primary diagnosis at index admission (psychotic disorders, personality disorders, other eligible disorders); and first Language (English, other). A similar post-hoc subgroup analysis was done for gender (female, male). The CACE was estimated with a two-stage estimation procedure. In the first stage, a logistic regression of treatment receipt regressed on randomisation was conducted. In the second stage, a Poisson regression of the outcome on treatment receipt was conducted. The analysis was adjusted for the same covariates as the ITT analysis. A bootstrap (1000 samples) was used to obtain bias corrected and accelerated confidence intervals. Because the standard error is estimated with a bootstrap, we only provide confidence intervals and not p values. The paramed command in Stata was used to estimate the CACE, according to procedures outlined by Dunn and colleagues.^30^ Time-to-first readmission curves by treatment allocation for the 12 months post-discharge were presented to observe the cumulative rate of readmission. Prespecified sensitivity analyses for the 4-month secondary outcomes, MANSA, BPRS, HHI, and SIX, were conducted under the missing not at random assumption over a range of plausible scenarios to assess how robust the results were to departures from missing at random on the treatment estimates. Time-to-first readmission curves by treatment allocation within 12 months post-discharge were presented to observe the cumulative rate of readmission over the 12 months post-discharge. A planned process measure presented the total number of community mental health service appointments that were not attended as a proportion of the total number of appointments scheduled, by treatment allocation, during both the 12 months before and the 12 months after the index admission. A post-hoc analysis presented the number and proportion of patients readmitted to psychiatric inpatient care in the 12 months post-discharge by ethnicity and by treatment allocation.

The trial is registered with the ISRCTN registry, ISRCTN10043328, and was overseen by an independent steering committee and a data monitoring committee.

### Role of the funding source

The funder of the study had no role in study design, data collection, data analysis, data interpretation, or writing of the report.

## Results

**Figure 1:**
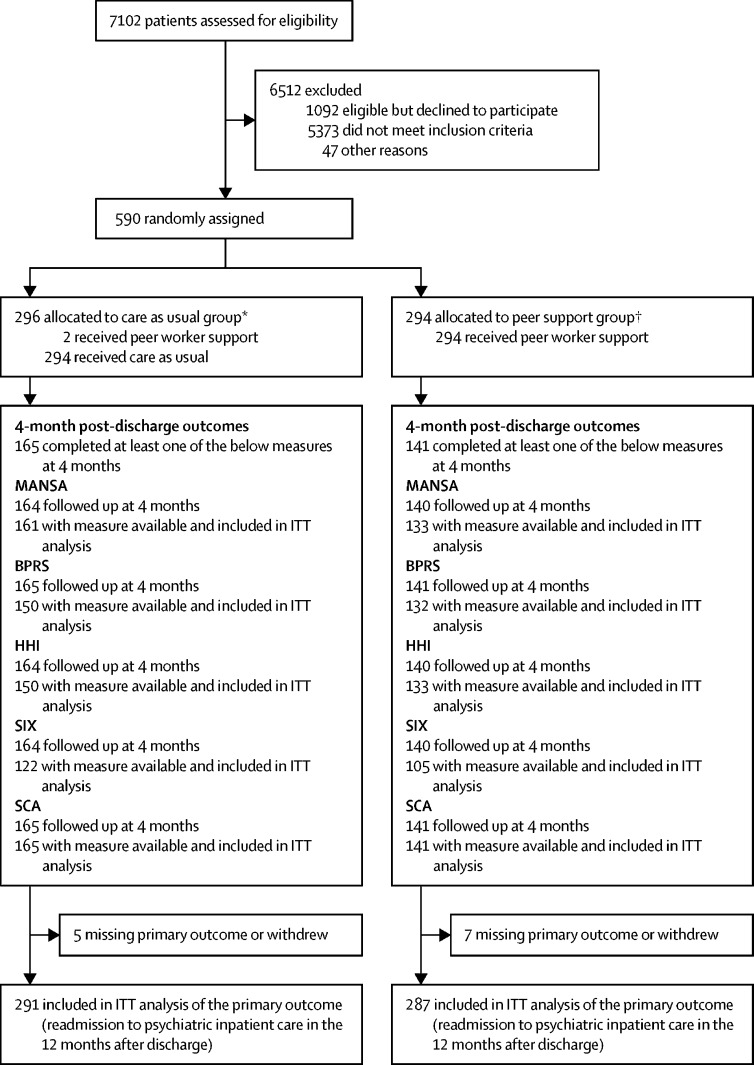
CONSORT flow chart of patients MANSA=Manchester Short Assessment of Quality of Life. ITT=intention to treat. BPRS=Brief Psychiatric Rating Scale. HHI=Herth Hope Index. SIX=Objective Social Outcomes Index. SCA=Social Contacts Assessment. *One ineligible patient was randomly assigned to care as usual (included in analyses) and one patient withdrew consent for use of data during the trial (excluded from all analyses). †Three patients withdrew consent for use of data during the trial (excluded from all analyses).

Between Dec 1, 2016, and Feb 8, 2019, of 7102 patients screened for the trial, 1682 were eligible, of whom 590 consented and were randomly assigned to groups (294 to peer support and 296 to care as usual; figure 1). Four patients withdrew their data from the study after group allocation, with 586 retained in the trial. One ineligible patient was randomised to care as usual and two patients in the care as usual group received the peer support intervention (all patients were analysed on an ITT basis in the group to which they were allocated). The 4-month interview was completed with at least one outcome measure recorded by 306 (52%) of 586 patients, and 584 patients had a linkable electronic record at the 12-month follow-up. 578 patients (287 in the peer support group and 291 in the care as usual group) were included in the final ITT analysis at 12 months (figure 1). Between Dec 1, 2016, and Feb 28, 2017, 31 of the patients were recruited into the internal pilot. Pilot data were analysed, presented to the steering committee, and a decision was made to continue the trial, with recruitment until Feb 8, 2019.

Baseline characteristics were well balanced between groups (table 1). Overall, among 578 patients with available gender data, 306 (53%) were women, 267 (46%) were men, three (1%) were transgender, and two (<1%) preferred not to say. 571 patients had available age data; mean age was 39·7 (SD 13·7; range 18–75) years. Of 576 patients with ethnicity data, 353 (61%) identified as of White ethnicity, 94 (16%) as Black, African, Caribbean, or Black British, 68 (12%) as Asian or Asian British, 48 (8%) as of mixed or multiple ethnic groups, and 13 (2%) as of other ethnic groups. 263 (45%) of the total 586 patients had a diagnosis of schizophrenia, schizotypal, or delusional disorder (table 1). Severity of psychiatric symptoms, as measured with the BPRS, was similar in both groups, with a mean score of 31·7 (SD 10·7) in the care as usual group, and 29·5 (9·6) in the peer support group. Psychiatric hospitalisation within the previous 2 years was an inclusion criterion and was fulfilled by all patients; 399 (68%) of 584 patients with available data had at least one hospitalisation within the 12 months before the index admission (appendix p 1). The median number of days in hospital for all 584 patients with available data was 16 days (IQR 0–42) in the care as usual group and 14 days (0–39) in the peer support group.

**Table 1:** Baseline characteristics

		**Number of participants with available data**		**Summary measure**	
		Care as usual* (n=296)	Peer support† (n=294)	Care as usual	Peer support
Gender		292 (99%)	286 (97%)	..	..
	Female	..	..	159 (54%)	147 (51%)
	Male	..	..	130 (45%)	137 (48%)
	Transgender	..	..	2 (1%)	1 (<1%)
	Prefer not to say	..	..	1 (<1%)	1 (<1%)
Age, years		291 (98%)	280 (95%)	..	..
	Mean	..	..	40·0 (13·1)	39·4 (14·2)
	Median	..	..	38 (31–50)	38 (27–51)
Sexual orientation		290 (98%)	286 (97%)	..	..
	Bisexual	..	..	26 (9%)	20 (7%)
	Gay	..	..	6 (2%)	10 (3%)
	Heterosexual	..	..	232 (80%)	239 (84%)
	Lesbian	..	..	5 (2%)	5 (2%)
	Not completed/Declined to answer	..	..	21 (7%)	12 (4%)
Diagnostic group		295 (>99%)	291 (99%)	..	..
	Schizophrenia, schizotypal, and delusional disorders (ICD-10 diagnosis F20–29)	..	..	134 (45%)	129 (44%)
	Specific personality disorders (ICD-10 diagnosis F60)	..	..	61 (21%)	58 (20%)
	Other eligible non-psychotic disorders (excluding ICD-10 diagnoses F00–09, F10–19, F50, and F70–79)	..	..	100 (34%)	104 (36%)
First language		288 (97%)	280 (95%)	..	..
	English	..	..	243 (84%)	226 (81%)
	Other	..	..	45 (16%)	54 (19%)
Ethnicity		293 (99%)	283 (96%)	..	..
	Asian or Asian British	..	..	32 (11%)	36 (13%)
	Black, African, Caribbean, or Black British	..	..	48 (16%)	46 (16%)
	Mixed or multiple ethnic groups	..	..	18 (6%)	30 (11%)
	Other ethnic group	..	..	5 (2%)	8 (3%)
	White	..	..	190 (65%)	163 (58%)
Manchester Short Assessment of Quality of Life‡		292 (99%)	283 (96%)	..	..
	Mean	..	..	3·9 (1·1)	4·2 (1·1)
	Median	..	..	4·0 (3·2–4·7)	4·2 (3·4–4·8)
Objective Social Outcomes Index§		251 (85%)	244 (83%)	..	..
	Mean	..	..	3·2 (1·3)	3·2 (1·3)
	Median	..	..	3 (2–4)	3 (2–4)
Herth Hope Index¶		280 (95%)	275 (94%)	..	..
	Mean	..	..	33·1 (7·9)	33·1 (8·1)
	Median	..	..	34 (28–39)	34 (28–39)
Brief Psychiatric Rating Scale‖		268 (91%)	272 (93%)	..	..
	Mean	..	..	36·6 (10·2)	34·5 (9·9)
	Median	..	..	36 (29–43)	33 (27–41)

Data are n (%), mean (SD), or median (IQR). Percentages might not always add to 100% due to rounding.

* One patient withdrew consent for use of data in the care as usual group after randomisation; this patient is among those with missing data throughout the table.

† Three patients withdrew consent for use of data in the peer support group after randomisation; these patients are among those with missing data throughout the table.

‡ Score range 1–7, higher scores indicate better quality of life; up to two items are allowed to be missing to calculate the score.

§ Score range 0–6, higher scores indicate higher social inclusion.

¶ Score range 12–48, higher scores indicate higher hope for the future.

‖ Score range 0–126, higher scores indicate more severe symptoms.

The median duration of the index admission was 43 days (21–85) in the peer support group and 42 days (22–75) in the care as usual group. Of 258 admissions for which type was known, 133 (52%) were involuntary in the peer support group and 129 (51%) of 252 in the care as usual group. Overall, 474 (84%) of 566 patients with known discharge destination were discharged to their usual residence (appendix p 2). A total of 22 (4%) of 584 patients with known time of randomisation were randomly allocated after discharge from their index admission (appendix p 3). Four patients were still in hospital (one in the care as usual group, three in the peer worker group) following their index admission at the end of study, and for 78 (13%) of the 578 patients included in the final analysis (not including those who died or withdrew from the study), follow-up was curtailed to less than 12 months (median 331 days [288–351]) to allow sufficient time for data cleaning and analysis.

In the peer support group, 136 (47%) of 287 patients were readmitted to psychiatric inpatient care within 12 months after the index admission. 146 (50%) of 291 were readmitted in the care as usual group (table 2). The unadjusted risk difference was 0·03 (95% CI –0·11 to 0·05; p=0·51) in favour of the peer support group. The adjusted RR of readmission in the ITT analysis was 0·97 (95% CI 0·82–1·14; p=0·68), and the adjusted OR was 0·93 (95% CI 0·66–1·30; p=0·68).

**Table 2:** Primary and 4-month secondary outcomes

	**Number of participants with available data and included in analysis**		**Summary measure**		**Statistical measures**	
	Care as usual* (n=296)	Peer support† (n=294)	Care as usual	Peer support	Treatment effect (95% CI)	p value
**Primary outcome**						
Readmission to psychiatric inpatient care in the 12 months post-discharge‡	291 (98%)	287 (98%)	146 (50%)§	136 (47%)§	0·93 (0·66 to 1·30)¶	0·68
Readmission to psychiatric inpatient care in the 12 months post-discharge‡	291 (98%)	287 (98%)	146 (50%)§	136 (47%)§	0·97 (0·82 to 1·14)‖	0·68
Readmission to psychiatric inpatient care in the 12 months post-discharge‡	291 (98%)	287 (98%)	146 (50%)§	136 (47%)§	−0·03% (−0·11 to 0·05)**	0·51
**Secondary outcomes**						
Manchester Short Assessment of Quality of Life	161 (54%)	133 (45%)	4·1 (1·0)††	4·4 (0·9)††	0·17 (−0·01 to 0·36)‡‡	0·07
Brief Psychiatric Rating Scale	150 (51%)	132 (45%)	31·7 (10·7)††	29·5 (9·6)††	−0·59 (−2·70 to 1·52)‡‡	0·58
Herth Hope Index	150 (51%)	133 (45%)	32·3 (7·2)††	33·8 (7·0)††	0·50 (−0·80 to 1·79)‡‡	0·45
Objective Social Outcomes Index	122 (41%)	105 (36%)	3·2 (1·0)††	3·2 (1·0)††	0·10 (−0·13 to 0·34)‡‡	0·38
Social Contacts Assessment, number of contacts	165 (56%)	141 (48%)	3 (1–4)§§	2 (1–5)§§	1·07 (0·85 to 1·34)¶¶	0·56

* One patient withdrew consent for use of data in the care as usual group after randomisation; this patient is among those with missing data throughout the table.

† Three patients withdrew consent for use of data in the peer support group after randomisation; these patients are among those with missing data throughout the table.

‡ Model taking into account clustering did not converge and hence a logistic regression model was fitted ignoring clustering.

§ Number (%).

¶ Adjusted odds ratio.

‖ Adjusted risk ratio.

** Unadjusted risk difference.

†† Mean (SD).

‡‡ Adjusted mean difference.

§§ Median (IQR).

¶¶ Adjusted rate ratio.

In the CACE analysis, the risk of readmission for those who received peer support (RR 0·88 [0·76–0·99]) was lower than the value obtained from the ITT analysis. In the peer support group, 163 (62%) of 265 patients with available contact data (appendix p 10) had had at least two sessions of peer support, at least one of which was post-discharge, and these patients were included in the CACE analysis as the group who received peer support (appendix p 4). In subgroup analyses, for patients of any Black ethnicity, the adjusted OR of readmission was 0·40 (0·17–0·94), while for any other ethnicity the OR was 1·12 (0·77–1·63; interaction p=0·031). No other subgroup effects were found (appendix p 5).

Observed differences in the secondary outcomes collected at 4 months were small and none were statistically significant (table 2). Given the likelihood of incomplete 4-month questionnaire data, prespecified sensitivity analyses were performed for a range of scenarios to assess departures from the missing at random assumption (appendix pp 6–9). The estimates from the models for each outcome were not robust to departures from this assumption, limiting the confidence with which we might interpret these findings. At least part of the 4-month follow-up occurred outside the period specified in the protocol for 27 (9%) of 306 patients (appendix p 3). Assessors were unmasked by 52 (17%) of 306 patients (38 in the peer support group, 14 in the care as usual group) who revealed their allocation during collection of secondary outcome data at 4 months.

**Figure 2:**
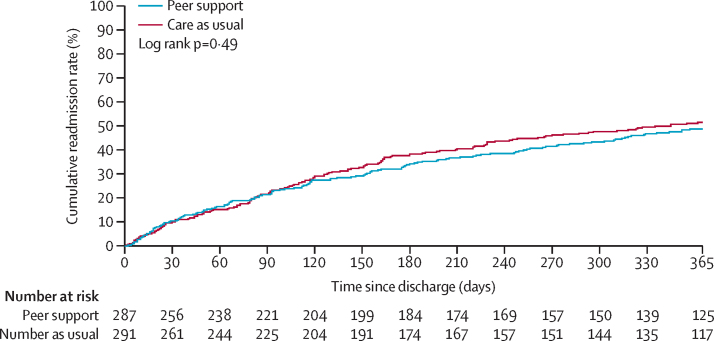
Time-to-first readmission by treatment allocation within 12 months post-discharge

We observed no statistically significant differences between the peer support and care as usual groups in any of the secondary outcomes assessed at 12 months (table 3). Time-to-first readmission curves within 12 months post-discharge were similar between the groups (figure 2).

**Table 3:** 12-month secondary outcomes

	**Number of participants with available data and included in analysis**		**Summary measure**		**Statistical measures**	
	Care as usual* (n=296)	Peer support† (n=294)	Care as usual	Peer support	Treatment effect (95% CI)	p value
Number of readmissions to psychiatric inpatient care in the 12 months post-discharge	291 (98%)	287 (98%)	1 (1–2)‡	1 (1–2)‡	0·95 (0·75–1·19)§	0·64
Number of voluntary admissions to psychiatric inpatient care in the 12 months post-discharge¶	253 (85%)	257 (87%)	1 (0–1)‡	1 (0–2)‡	1·07 (0·77–1·48)§	0·71
Number of involuntary admissions to psychiatric inpatient care in the 12 months post-discharge¶	253 (85%)	257 (87%)	1 (0–1)‡	1 (0–1)‡	0·88 (0·64–1·22)§	0·44
Total length of stay for all readmissions, days¶	291 (98%)	287 (98%)	57 (27–128)‡	61 (26–99)‡	0·81 (0·51–1·29)§	0·38
Number of separate episodes of liaison psychiatry contact in hospital accident and emergency¶	291 (98%)	287 (98%)	0 (0–1)‖	0 (0–1)‖	1·18 (0·84–1·66)§	0·34
Number of crisis resolution and home treatment team contacts¶	291 (98%)	287 (98%)	3 (0–13)‖	2 (0–16)‖	0·90 (0·66–1·23)§	0·53
Time to first readmission to psychiatric inpatient care, days¶	291 (98%)	287 (98%)	107 (46–180)‡	104 (36–201)‡	0·95 (0·75–1·20)**	0·66

* One patient withdrew consent for use of data in the care as usual group after randomisation; this patient is among those with missing data throughout the table.

† Three patients withdrew consent for use of data in the peer support group after randomisation; these patients are among those with missing data throughout the table.

‡ Median (IQR) provided only for those who had a readmission ie, 146 in care as usual group and 136 in peer support group.

§ Adjusted rate ratio.

¶ Model taking into account clustering did not converge and hence a logistic regression model was fitted ignoring the clustering.

‖ Median (IQR).

** Adjusted hazard ratio.

A total of 31 peer workers provided peer support, one of whom did not record their contacts with patients. The 30 peers with recorded contacts were assigned a mean of 9 patients (SD 7·6), ranging from one to 39 each. Adherence to the intervention was assessable in 268 (91%) of 294 patients before discharge and 265 patients (90%) after discharge. A mean of 1·8 face-to-face contacts (SD 2·9) with a peer worker took place in hospital, and 4·4 (4·6) after discharge. Mean total time spent in face-to-face peer worker sessions post-discharge was 550 min (SD 449). Mean number of telephone contacts was 0·4 (SD 1·0) in hospital and 2·1 (3·7) post-discharge (appendix p 10).

A total of 67 serious adverse events were reported in the trial (34 in the peer support group, 33 in the care as usual group), in 51 (9%) of 586 patients retained in the study after withdrawals (26 in the peer support group, 25 in the care as usual group). 41 serious adverse events occurred in women (n=32), 26 occurred in men (n=19), and none occurred in the transgender and unspecified gender groups. One serious adverse event in the peer support group, an incident of self-harm, was reported as unexpected and related to the intervention. The serious adverse events (table 4) included 12 deaths (12 [2%] of 586 patients), none of which were reported as related to the study. Threat to life (self-harm) was the most common serious adverse event (35 [52%] of 67 serious adverse events in 28 patients). 391 other adverse events were also reported in 146 (25%) of 586 patients, with physical assault or threatening behaviour the most common (93 [28%] of 391 adverse events in 52 patients; appendix p 14). 220 adverse events occurred in women (n=75), 162 occurred in men (n=67), nine occurred in transgender patients (n=4), and none occurred in patients of unspecified gender.

**Table 4:** Serious adverse events

		**Summary measure**	
		Care as usual (n=295)*	Peer support (n=291)†
Participants with ≥1 event		25 (8%)	26 (9%)
Total number of events		33	34
Number of unexpected events related to the intervention		NA	1 (0·3)
Type of event			
	Death	6	6
	Life threatening (not self-harm)	3	4
	Hospitalisation or prolongation of existing hospitalisation	3	2
	Persistent or significant disability or incapacity	0	1
	Threat to life (self-harm)	18	17
	Other‡	3	4

* One patient withdrew consent for use of data in the care as usual group.

† Three patients withdrew consent for use of data in the peer worker group.

‡ These seven events were all absconds from care.

Community mental health service appointments that were not attended as a proportion of total community mental health service appointments, by treatment allocation, in the 12 months before and after index admission are presented in the appendix (p 10). Readmission rates among ethnic groups by treatment allocation are presented in the appendix (p 11).

## Discussion

This large-scale trial provided an overall clear and consistent result; one-to-one peer support in addition to care as usual did not have a significant effect on psychiatric readmission. There were neither a significant reduction in readmission as the primary outcome, nor significant effects on any secondary outcome, including number of readmissions, days in hospital, time to (first) readmission, or emergency or crisis service use in the year after discharge. Symptom severity was not reduced and psychosocial outcomes were not improved after 4 months. Two pre-specified analyses suggested a benefit for subgroups. A CACE analysis indicated that patients in the peer support group who received a pre-defined minimal amount of the intervention were less likely to be readmitted than patients in the control group who, according to the analysis, would also have received the minimal amount of peer support if such support had been offered to them. Additionally, compared with the care as usual group, patients of any Black ethnicity in the peer support group were significantly less likely to be readmitted than patients of any other ethnicity.

Considering the shortcomings of existing trials of peer support to date,^17^, ^18^ our trial has important strengths. These include robust procedures for concealment of allocation from assessors, complete reporting of outcomes, and low attrition at the primary endpoint. Completeness of primary outcome and most secondary outcome data on the use of health-care services at 12 months was 98%, thus adding power to analyses given that we had anticipated 90%. Conversely, our sample size calculation had assumed a readmission rate in the population of 34%; the observed readmission rate of nearly 50% overall therefore resulted in some reduction in statistical power. However, we note that our results are consistently negative with the sample size target having been met, resulting in a robust primary analysis.

Although the data obtained from clinical records were complete for most patients, we were unable to interview around 48% of patients face-to-face at the 4-month follow up. A study of continuity of psychiatric care post-discharge encountered similar difficulties,^31^ suggesting that face-to-face follow-up, in the community, of people recruited as psychiatric inpatients is generally challenging.

The study reflects findings from a recent systematic review of one-to-one peer support in a range of mental health service settings that suggested, on the basis of pooled data from trials to date, peer support is unlikely to have an effect on psychiatric hospital admission, length of stay in hospital or clinical severity.^16^ The review did indicate a modest positive effect of one-to-one peer support on self-reported recovery and empowerment, although these outcomes were not measured in our trial, leaving open the possibility that peer support has psychosocial benefits for patients in some settings. We note that a large proportion of patients assessed for eligibility did not meet our inclusion criteria (figure 1). Thus, our study does not preclude the possibility that patients at first admission or from excluded diagnostic groups might benefit from peer support.

Engagement in the peer support intervention was low, with a mean of 1·8 face-to-face contacts (SD 2·9) per patient before discharge in the peer support group, and 4·4 contacts (4·6) after discharge (compared with a total planned 14 contacts). The findings of the CACE analysis suggest that the 163 (62%) patients (of 265 with post-discharge contact data) who had improved engagement might have benefitted from the intervention. The effect estimate of this difference is small, and the finding does not change the overall result that offering peer support at discharge did not have a significant benefit. The finding does, however, suggest a link between increased engagement and improved outcomes and raises the question as to why engagement with the intervention was not more complete even though recruitment, training, and supervision of peer workers were all implemented as planned. At least two reasons might explain this.

First, many patients might not have had the opportunity to establish a good relationship with the peer worker. The model underpinning our intervention presumes that peer support begins with building trusting relationships on the basis of shared lived experience^20^, ^21^ and the assumption was that this would happen while the patient was still in the hospital. Discharge from hospital treatment can take place at short notice, and patients might not have had enough time and occasions to build relationships with their peer workers before discharge. We also note that choice and control over engaging with peer support has been identified as a key principle underlying peer support.^20^ As such, peer support is an offer rather than a prescription and some patients might have felt that the peer worker they were assigned was not the right person for them. In the context of the trial we had no opportunity to enable participants to express a preference over peer worker.

Second, we might have targeted a group of patients who found engaging with peer support particularly hard at a difficult point in the care pathway, or whom peer workers found challenging to support. Our population was at high risk of readmission, being inpatients with a history of psychiatric admission in the preceding two years,^4^ reflected in the higher than anticipated number of readmissions (282 [49%] of 578 patients) across both treatment groups. Additionally, 399 (68%) of 584 patients with known readmission history in the previous 12 months had been admitted at least once in the year before recruitment, and 262 (51%) of 510 with known index admission type had been involuntarily admitted before recruitment. A recent, smaller trial of peer support for discharge, in which the whole sample had been involuntarily admitted, found no significant effect on any outcome assessed.^32^ In comparison, in a pilot trial evaluating peer support for discharge, only 21 (46%) of 46 patients randomised had been admitted in the year before recruitment, with just seven (30%) of 23 patients allocated to peer support involuntarily admitted before recruitment.^15^ Rates of previous admission and involuntary admission in trials of community-based peer support are considerably lower than in the present study,^33^, ^34^ leaving open the possibility that peer support might have a different effect on less at-risk groups. Involuntary admission has been shown to be associated with reduced adherence to community treatment.^35^, ^36^ Our intervention, assuming as it did a high degree of flexibility in the activities that took place, might not have been sufficiently structured either to maintain engagement or to meet the high level of need of our high-risk group of patients. A more structured intervention, as successfully delivered in a trial of peer supported self-management to reduce acute service use after discharge from crisis resolution and home treatment team care,^37^ might have been of increased benefit to this population.

We found that patients of any Black ethnicity receiving peer support were significantly less likely to be readmitted in the year post-discharge than those of any other ethnicity, compared with corresponding patients in the control group. Being of Black ethnicity is a predictor of psychiatric readmission,^38^ with Black people over-represented in acute inpatient care compared with the general population.^39^ The finding offers encouragement that peer support might be helpful for this group. However, the sample size for the subgroup analysis was small and further research should explore how peer support might be best implemented among Black people using mental health services, perhaps leading to a targeted intervention.

In conclusion, this study showed that one-to-one peer support, offered before discharge and continuing after discharge from inpatient psychiatric care in addition to care as usual, was not superior to care as usual alone. Although our findings seem to confirm research to date that suggests peer support at discharge does not have a significant effect on subsequent hospitalisation, days in hospital, or severity of symptoms, we note that our study was in a population with high needs at the point of hospital discharge. Although the need for good evidence of cost-effectiveness remains, the implication for policy is that one-to-one peer support for patients at risk of readmission should not be commissioned with the expectation that it will reduce readmission following discharge. Future studies might focus on identifying other patient groups and other aspects of patient care (eg, psychosocial) in which peer support could be beneficial, and on further developing peer support programmes to improve engagement and outcomes.

## Data sharing

Individual participant data that underlie the results reported in this Article, after de-identification, and the statistical analysis plan and analytical code will be made available following publication, on reasonable request, to researchers who provide a methodologically sound proposal. Proposals should be directed to the corresponding author; to gain access, data requestors will need to sign a data access agreement.

## Declaration of interests

We declare no competing interests.
