# Supplementary material for: Peer support for discharge from inpatient mental health care versus care as usual in England (ENRICH): a parallel, two-group, individually randomised controlled trial
Source: Lancet Psychiatry. 2022 Feb;9(2):125–36. doi: 10.1016/S2215-0366(21)00398-9 (PMC8776565; doi:10.1016/S2215-0366(21)00398-9)
Supplement: Supplementary appendix [file mmc1.pdf]

THE LANCET
Psychiatry

Supplementary appendix

This appendix formed part of the original submission and has been peer reviewed. We post it as supplied by the authors.

Supplement to: Gillard S, Bremner S, Patel A, et al. Peer support for discharge from inpatient mental health care versus care as usual in England (ENRICH): a parallel, two-group, individually randomised controlled trial. *Lancet Psychiatry* 2022; **9:** 125–36.

Supplementary table 1 – Pre-index admission characteristics

Pre-index Admission Characteristics (12 months prior to index admission)	Number of participants with available data - no. (%)		Summary measure	
	Care as usual (n=296)	Peer worker (n=294)	Care as usual	Peer worker
Number of admissions to psychiatric inpatient care - no. (%)	293 (99.0)	291 (99.0)		
0			84 (28.7)	101 (34.7)
1			144 (49.1)	130 (44.7)
2			39 (13.3)	35 (12.0)
3 or more			26 (8.9)	25 (8.6)
Number of voluntary admissions - no. (%)	255 (86.1)	258 (87.8)		
0			158 (62.0)	166 (64.3)
1			69 (27.1)	56 (21.7)
2 or more			28 (11.0)	36 (14.0)
Number of compulsory admissions - no. (%)	255 (86.1)	258 (87.8)		
0			154 (60.4)	161 (62.4)
1			81 (31.8)	79 (30.6)
2 or more			20 (7.8)	18 (7.0)
Total length of stay over all admissions (calendar days)	293 (99.0)	291 (99.0)		
Mean (SD)			32.7 (48.0)	28.9 (41.3)
Median (IQR)			16 (0-42)	14 (0-39)
Number of A&E attendances - no. (%)	293 (99.0)	291 (99.0)		
0			123 (42.0)	113 (38.8)
1			64 (21.8)	75 (25.8)
2			37 (12.6)	43 (14.8)
3 or more			69 (23.5)	60 (20.6)
Number of crisis resolution or home treatment team contacts - no. (%)	293 (99.0)	291 (99.0)		
0			72 (24.6)	86 (29.6)
1			45 (15.4)	30 (10.3)
2			15 (5.1)	14 (4.8)
3 or more			161 (54.9)	161 (55.3)

Abbreviations: SD, standard deviation; IQR, Interquartile range

Supplementary table 2 – Index admission characteristics

Index Admission Characteristics	Number of participants with available data - no. (%)		Summary measure	
	Care as usual (n=296)	Peer worker (n=294)	Care as usual	Peer worker
Length of stay (calendar days)	293 (99.0)	291 (99.0)		
Mean (SD)			73.8 (122.9)	82.7 (132.2)
Median (IQR)			42 (22-75)	43 (21-85)
Type of admission - no. (%)	252 (85.1)	258 (87.8)		
Voluntary			123 (48.8)	125 (48.4)
Compulsory			129 (51.2)	133 (51.6)
Discharge destination - no. (%)	286 (96.6)	280 (95.2)		
Usual place of residence			239 (83.6)	235 (83.9)
Temporary place of residence			37 (12.9)	36 (12.9)
Local authority residential home			2 (<1)	2 (<1)
Other			8 (2.8)	7 (2.5)

Abbreviations: SD, standard deviation; IQR, Interquartile range

Supplementary table 3 – Protocol deviations

Protocol Deviation - no. (%)	Number of participants with available data - no. (%)		Summary measure	
	Care as usual (n=296)	Peer worker (n=294)	Care as usual	Peer worker
4-month researcher follow-up completed outside 120-180 days post-discharge	165 (55.7)	141 (48.0)	12 (7.3)	10 (7.1)
4-month participant follow-up completed outside 120-180 days post-discharge	164 (55.4)	140 (47.6)	12 (7.3)	14 (10.0)
Randomised and subsequently found not to meet inclusion criteria	295 (99.7)	291 (99.0)	1 (0.3)	0 (0.0)
Randomised after discharge from index admission	293 (99.0)	291 (99.0)	7 (2.4)	15 (5.2)
Participant in care as usual group received peer worker	295 (99.7)	N/A	2 (0.7)	N/A
Participant in peer worker group received care as usual	N/A	291 (99.0)	N/A	0 (0.0)

Supplementary table 4 – CACE Analysis.

	Risk Ratio (95% CI)
**Natural indirect effect**	0.88 (0.76, 0.99)
**Controlled direct effect**	1.10 (0.89, 1.35)
**Natural direct effect**	1.10 (0.89, 1.35)
**Marginal total effect**	0.96 (0.80, 1.14)

Notes:

Participants were classified as receiving the intervention if they have had at least two sessions with their peer worker, at least one of which was post discharge from hospital. A two-stage estimation approach was used. In the first stage, a logistic regression of treatment receipt on randomisation was conducted including 560 cases with treatment receipt and covariates recorded. In the second stage, a Poisson regression regressing the outcome on treatment receipt was conducted including 556 cases with both the primary outcome and treatment receipt variable recorded. This was implemented in Stata using the 'paramed' command. A bootstrap (1000 samples) was used to obtain bias corrected and accelerated confidence intervals. (Dunn, G., Emsley, R., Liu, H., Landau, S., Green, J., White, I., & Pickles, A. Evaluation and validation of social and psychological markers in randomised trials of complex interventions in mental health: a methodological research programme. Health technology assessment 2008; 19(93): 1-116.

The *natural indirect effect* estimates the effect on outcome of participating in peer support and is the key result from this analysis. The controlled direct effect is the estimate of the direct path between randomisation and outcome. Similarly, the natural direct effect can be understood as an estimate of the effect of randomisation when nobody in the trial has peer support (the mediator is fixed at zero). These two estimates are identical. The marginal total effect is the composite effect.

Supplementary table 5 – Subgroup Analyses

Subgroup Variable	Number of participants with available data and included in analysis - no. (%)		Readmission to psychiatric inpatient care in the 12 months post-discharge - no. (%)		Adjusted odds ratio (95% CI)	P-value for interaction
	Care as usual[a] (n=295/296)	Peer worker[b] (n=291/294)	Care as usual	Peer worker		
Ethnicity[c, d]						
Black/African/Caribbean/Black British	48/48 (100.0)	46/46 (100.0)	28/48 (58.3)	17/46 (37.0)	0.40 (0.17, 0.94)	0.03
Any other ethnicity	241/245 (98.4)	233/237 (98.3)	117/241 (48.5)	117/233 (50.2)	1.12 (0.77, 1.63)	
Diagnostic Group[d]						
F20-29 (Schizophrenia, schizotypal and delusional disorders)	132/134 (98.5)	128/129 (99.2)	65/132 (49.2)	54/128 (42.2)	0.79 (0.48, 1.31)	0.67
F60 (Specific personality disorders)	59/61 (96.7)	56/58 (96.6)	35/59 (59.3)	32/56 (57.1)	0.98 (0.45, 2.11)	
Other eligible non-psychotic disorders	100/100 (100.0)	103/104 (99.0)	46/100 (46.0)	50/103 (48.5)	1.11 (0.63, 1.95)	
First language[d, e]						
English	240/243 (98.8)	222/226 (98.2)	124/240 (51.7)	105/222 (47.3)	0.88 (0.60, 1.28)	0.31
Other	44/45 (97.8)	54/54 (100.0)	19/44 (43.2)	28/54 (51.9)	1.42 (0.62, 3.23)	
Gender[d, f]						
Female	157/159 (98.7)	144/147 (98.0)	79/157 (50.3)	74/144 (51.4)	1.05 (0.66, 1.68)	0.57
Male	128/130 (98.5)	136/137 (99.3)	64/128 (50.0)	61/136 (44.9)	0.86 (0.52, 1.42)	

[a] 1 patient withdrew consent for use of data in the care as usual group

[b] 3 patients withdrew consent for use of data in the peer worker group

[c] Subgroup analysis conducted only on complete ethnicity data and complete outcome data. In addition, 2 participants in the care as usual group were missing ethnicity data and 8 participants in the peer worker group were missing ethnicity data and therefore denominators do not add up to 295 (care as usual) and 291 (peer worker)

[d] Model taking into account clustering did not converge and hence a logistic regression model was fitted ignoring clustering

[e] In addition**,** 7 participants in the care as usual group were missing first language data and 11 participants in the peer worker group were missing first language data and therefore denominators do not add up to 295 (care as usual) and 291 (peer worker)

[f] This subgroup analysis was conducted post-hoc and only for males and females. The denominators will not add up to 295 (care as usual) and 291 (peer worker) due to missing data for gender and restricting the analysis to only males and females as the proportions for transgender and prefer not say were low.

Supplementary table 6 – Sensitivity analysis (MANSA)

Assumed mean response for participants with missing data in the care as usual group	Assumed mean response for participants with missing data in the peer worker group	Treatment effect (95% CI)
2	1	-0.19 (-0.38, -0.01)
2	2	0.35 (0.16, 0.53)
2	3	0.89 (0.71, 1.08)
3	2	-0.11 (-0.29, 0.08)
3	3	0.44 (0.25, 0.62)
3	4	0.98 (0.79, 1.17)
4	3	-0.02 (-0.20, 0.17)
4	4	0.53 (0.34, 0.71)
4	5	1.07 (0.88, 1.25)
5	4	0.07 (-0.11, 0.26)
5	5	0.61 (0.43, 0.80)
5	6	1.16 (0.97, 1.34)
6	5	0.16 (-0.03, 0.35)
6	6	0.70 (0.52, 0.89)
6	7	1.25 (1.06, 1.43)

This analysis excluded all participants who did not have a complete score at 4-months. This analysis in Table 2 assumed the excluded participants were *missing-at-random* – that is, the reason these participants' data was missing was based upon variables that were included in the analysis.

The analyses in Supplementary Table 6 (above) have made different assumptions regarding participants who were excluded from the analysis in order to assess how robust the primary analysis results are to departures from the *missing-at-random* assumption. Specifically, the analyses presented in Supplementary Table 6 have assumed the excluded participants were *missing-not-at-random* – that is, the reason these participants' data were missing was actually based upon their MANSA scores at 4 months (e.g. participants with a lower MANSA score at 4 months were more likely to be excluded from the analysis).

Interpretation of Supplementary Table 6:
The results from Supplementary Table 6 indicate that the analysis of MANSA reported in Table 2 is not robust to departures from the missing-at-random assumption which is not surprising due to the high levels of missing data.

Supplementary table 7 – Sensitivity analysis (BPRS)

Assumed mean response for participants with missing data in the care as usual group	Assumed mean response for participants with missing data in the peer worker group	Treatment effect (95% CI)
20	0	-10.42 (-12.53, -8.31)
20	10	-4.96 (-7.07, -2.85)
20	20	0.51 (-1.60, 2.62)
20	30	5.97 (3.86, 8.08)
20	40	11.43 (9.32, 13.54)
40	20	-9.32 (-11.43, -7.21)
40	30	-3.86 (-5.97, -1.75)
40	40	1.60 (-0.51, 3.71)
40	50	7.07 (4.96, 9.18)
40	60	12.53 (10.42, 14.64)
60	40	-8.23 (-10.34, -6.12)
60	50	-2.76 (-4.87, -0.65)
60	60	2.70 (0.59, 4.81)
60	70	8.16 (6.05, 10.28)
60	80	13.63 (11.52, 15.74)
80	60	-7.13 (-9.24, -5.02)
80	70	-1.67 (-3.78, 0.45)
80	80	3.80 (1.69, 5.91)
80	90	9.26 (7.15, 11.37)
80	100	14.73 (12.62, 16.84)
100	80	-6.03 (-8.14, -3.92)
100	90	-0.57 (-2.68, 1.54)
100	100	4.90 (2.78, 7.01)
100	110	10.36 (8.25, 12.47)
100	120	15.82 (13.71, 17.93)

Interpretation of Supplementary Table 7:

The results from Supplementary Table 7 indicate that the analysis of BPRS reported in Table 2 is not robust to departures from the missing-at-random assumption which is not surprising due to the high levels of missing data.

Supplementary table 8 – Sensitivity analysis (Herth Hope Index)

Assumed mean response for participants with missing data in the care as usual group	Assumed mean response for participants with missing data in the peer worker group	Treatment effect (95% CI)
18	12	-1.84 (-3.13, -0.54)
18	15	-0.21 (-1.50, 1.09)
18	18	1.42 (0.13, 2.71)
18	21	3.05 (1.76, 4.34)
18	24	4.68 (3.39, 5.97)
24	18	-1.53 (-2.82, -0.23)
24	21	0.10 (-1.19, 1.39)
24	24	1.73 (0.44, 3.02)
24	27	3.36 (2.07, 4.65)
24	30	4.99 (3.69, 6.28)
30	24	-1.22 (-2.51, 0.07)
30	27	0.41 (-0.88, 1.70)
30	30	2.04 (0.74, 3.33)
30	33	3.67 (2.37, 4.96)
30	36	5.30 (4.00, 6.59)
36	30	-0.91 (-2.20, 0.38)
36	33	0.72 (-0.58, 2.01)
36	36	2.35 (1.05, 3.64)
36	39	3.98 (2.68, 5.27)
36	42	5.60 (4.31, 6.90)
42	36	-0.60 (-1.90, 0.69)
42	39	1.03 (-0.27, 2.32)
42	42	2.66 (1.36, 3.95)
42	45	4.28 (2.99, 5.58)
42	48	5.91 (4.62, 7.21)

Interpretation of Supplementary Table 8:

The results from Supplementary Table 8 indicate that the analysis of Herth Hope Index reported in Table 2 are not robust to departures from the missing-at-random assumption which is not surprising due to the high levels of missing data.

Supplementary table 9 – Sensitivity analysis (SIX)

Assumed mean response for participants with missing data in the care as usual group	Assumed mean response for participants with missing data in the peer worker group	Treatment effect (95% CI)
1	0	-0.48 (-0.71, -0.25)
1	1	0.16 (-0.08, 0.39)
1	2	0.80 (0.56, 1.03)
2	1	-0.43 (-0.66, -0.20)
2	2	0.21 (-0.02, 0.44)
2	3	0.85 (0.62, 1.08)
3	2	-0.38 (-0.61, -0.14)
3	3	0.26 (0.03, 0.50)
3	4	0.90 (0.67, 1.13)
4	3	-0.32 (-0.56, -0.09)
4	4	0.32 (0.08, 0.55)
4	5	0.95 (0.72, 1.19)
5	4	-0.27 (-0.50, -0.04)
5	5	0.37 (0.14, 0.60)
5	6	1.01 (0.78, 1.24)

Interpretation of Supplementary table 9:
The results from Supplementary Table 9 indicate that the analysis of SIX reported in Table 2 is not robust to departures from the missing-at-random assumption which is not surprising due to the high levels of missing data.

Supplementary table 10 – Adherence to intervention

Adherence to intervention	Number of participants with available data - no. (%)	Summary measure
	Peer worker (n=294)	Peer worker
Number of face to face contacts pre-discharge	268 (91.2)	
Mean (SD)		1.8 (2.9)
Median (IQR)		1 (0-2)
Number of face to face contacts post-discharge	265 (90.1)	
Mean (SD)		4.4 (4.6)
Median (IQR)		3 (0-8)
Number of telephone contacts pre-discharge	268 (91.2)	
Mean (SD)		0.4 (1.0)
Median (IQR)		0 (0-0)
Number of telephone contacts post-discharge	265 (90.1)	
Mean (SD)		2.1 (3.7)
Median (IQR)		1 (0-3)
Total length of time spent in face to face peer worker sessions post-discharge (minutes)	174/265 (65.7)	
Mean (SD)		550.4 (449.4)
Median (IQR)		458 (180-834)

Abbreviations: SD, standard deviation; IQR, Interquartile range

Supplementary table 11 – Process measures

Process Measure	Number of participants with available data - no. (%)		Summary measure No. of DNA/ no. of appointments (%)	
	Care as usual (n=296)	Peer worker (n=294)	Care as usual	Peer worker
Total number of DNA community mental health service appointments as a proportion of the total number of appointments scheduled during the 12 months prior to the index admission	293 (99.0)	291 (99.0)	916/7313 (12.5)	964/6997 (13.8)
Total number of DNA community mental health service appointments as a proportion of the total number of appointments scheduled during the 12 months post discharge from index admission	291 (98.3)	287 (97.6)	852/8628 (9.9)	922/7410 (12.4)

Abbreviation: DNA, Did Not Attend

Supplementary table 12 – Readmission rates among ethnic groups by treatment allocation

Variable	Readmission to psychiatric inpatient care in the 12 months post-discharge - no. (%)	
	Care as usual	Peer worker
Ethnicity		
Asian/Asian British	13/32 (40.6)	19/36 (52.8)
Black/African/Caribbean/Black British	28/48 (58.3)	17/46 (37.0)
Mixed/Multiple Ethnic Groups	10/18 (55.6)	12/30 (40.0)
Other Ethnic Group	4/4 (100.0)	4/8 (50.0)
White	90/187 (48.1)	82/159 (51.6)

List of study sites

Birmingham and Solihull Mental Health NHS Foundation Trust, Birmingham, UK

Bradford District Care NHS Foundation Trust, Bradford, UK

Central and North West London NHS Foundation Trust, London, UK

East London NHS Foundation Trust, London, UK

South West London and St George's Mental Health NHS Trust, London, UK

Surrey and Borders Partnership NHS Foundation Trust, Leatherhead, UK

Sussex Partnership NHS Foundation Trust, Worthing, UK

Supplementary table 13 – Adverse events

Event type	No. of events/no. of patients
Total adverse events	391/146
Absent without leave	58/41
Administrative error or complaint	7/7
Accident or injury	18/14
Distressed behaviour	15/14
Drug, alcohol or other contraband, possession or use	25/15
Fire setting, arson	7/7
Illness or medical	30/20
Intruder	1/1
Physical assault or threatening behaviour	93/52
Safeguarding incident	2/2
Self-harm (non-life threatening)	89/38
Sexual assault or sexually inappropriate behaviour	16/13
Theft or damage to property	4/3
Verbal assault or verbally threatening behaviour	25/18
